# Simplicity and Specificity in Language: Domain-General Biases Have Domain-Specific Effects

**DOI:** 10.3389/fpsyg.2015.01964

**Published:** 2016-01-12

**Authors:** Jennifer Culbertson, Simon Kirby

**Affiliations:** Language Evolution and Computation Research Unit, Linguistics and English Language, University of EdinburghEdinburgh, UK

**Keywords:** language evolution, domain-specificity, simplicity, typological universals, compositionality, word order, regularization

## Abstract

The extent to which the linguistic system—its architecture, the representations it operates on, the constraints it is subject to—is specific to language has broad implications for cognitive science and its relation to evolutionary biology. Importantly, a given property of the linguistic system can be “specific” to the domain of language in several ways. For example, if the property evolved by natural selection under the pressure of the linguistic function it serves then the property is domain-specific in the sense that its design is tailored for language. Equally though, if that property evolved to serve a different function or if that property is domain-general, it may nevertheless interact with the linguistic system in a way that is unique. This gives a second sense in which a property can be thought of as specific to language. An evolutionary approach to the language faculty might at first blush appear to favor domain-specificity in the first sense, with individual properties of the language faculty being specifically *linguistic* adaptations. However, we argue that interactions between learning, culture, and biological evolution mean any domain-specific adaptations that evolve will take the form of weak biases rather than hard constraints. Turning to the latter sense of domain-specificity, we highlight a very general bias, simplicity, which operates widely in cognition and yet interacts with linguistic representations in domain-specific ways.

## Introduction

One of the fundamental issues in cognitive science is the extent to which specifically linguistic mechanisms and representations underpin our knowledge of language and the way it is learned. This is in part because this issue has deep implications for the underlying uniqueness of a system we typically consider exclusive to humans. It has also been highly divisive in the sense that researchers from distinct traditions often have polar starting assumptions as to the likelihood of domain-specific properties of the language system. Here we will suggest that there are in fact (at least) two ways in which a given feature of the linguistic system may be considered to have domain-specific properties:

1. If that feature evolved by natural selection under the pressure of the linguistic function it serves.
2. If that feature is domain-general but interacts with the linguistic system and its representations in a way that is unique.

These two types of domain-specificity are quite different in terms of their implications for the evolution of language, and below we will discuss a set of results from computational models suggesting that domain-specificity of the first kind is unlikely to take the form of hard constraints on the linguistic system. Rather, if such constraints exist, they are likely to be weak biases, amplified through cultural evolution. This has important implications for linguistic theory, since, as we discuss below, many mainstream frameworks explicitly argue for hard domain-specific constraints and reject the notion of weak bias. The second type of domain-specificity, on the other hand, is likely to be widespread, and highlights the importance of collaborative efforts between experts in linguistic theory—who study the architecture and representations of language—and experts studying cognition across domains and species.

## Domain-specificity and evolution

In this section, we focus on the first sense of domain-specificity set out above, which interprets the issue in functional terms. This is perhaps the most obvious sense in which a particular aspect of the cognitive system might be specific to language, and it is the one which places a heavier burden on biological evolution. Importantly, it is the ultimate rather than proximate function that is relevant here; knowing that some feature of the cognitive system is used in processing or acquiring language is not, in and of itself, an argument for domain-specificity. We can no more argue that such a feature is language specific because it is active in language processing than we can argue for an aspect of cognition being chess-specific simply because it is active in the brain of a chess player. Rather, we need to consider the *ultimate* function of the cognitive architecture in question by looking to its evolutionary history. An aspect of our cognitive architecture is specific to language if it arose as an adaptive response to the problem of learning or using language^1^.

This argument places evolution right at the core of the question of the existence of language-specific features of our cognitive architecture. While some cross-species comparative data exist to help us trace the functional sources of various cognitive capacities (see Fitch, 2010 for review), these data are limited by the degree to which the relevant aspects of language are autapomorphies (completely novel traits that are not found in any other species). Recent research has turned to computational modeling to provide a more direct testing ground for specific hypotheses about how the capacities involved in language may have evolved. In particular, a number of papers have looked at whether domain-specific hard constraints on language can evolve from a prior stage where biases were less strong or not present at all (e.g., Kirby and Hurford, 1997; Briscoe, 2000; Smith and Kirby, 2008; Chater et al., 2009; Thompson, 2015). This is important, since many linguistic theories conceive of the language capacity as including a set of constraints of this kind: for example, Biberauer et al. (2014), working in the Minimalist framework (Chomsky, 1993), argue for a constraint which places a hard (inviolable) restriction on the distribution of the feature triggering movement (they call it the “Final-Over-Final” constraint, in a nod to the structural description of word orders the constraint rules out). Similarly, in Optimality Theory (Prince and Smolensky, 1993/2004), although a particular constraint may be violated in a given language, the standard mechanism for explaining typological data is to restrict the set of constraints. For example Culbertson et al. (2013) describe an OT grammar for word order in the noun phrase which completely rules out particular patterns by using a limited set of so-called alignment constraints (see also Steddy and Samek-Lodovici, 2011).

To investigate how hard domain-specific constraints of this type might evolve, Chater et al. (2009) describe a simulation of a population of language-learning agents. The genes of these agents specify whether learning of different aspects of language is tightly constrained or highly flexible. Agents in the simulation that successfully communicate are more likely to pass on their genes to future generations. The question that Chater et al. (2009) ask is whether genes encoding constraints evolve in populations which start out highly flexible under the selection pressure for communication. If they do, then this would support a language faculty in which language acquisition is constrained by domain-specific principles. This process, whereby traits that were previously acquired through experience become nativised, is known as the *Baldwin Effect* (Baldwin, 1896; Maynard Smith, 1986; Hinton and Nowlan, 1987), and a number of authors have suggested it played a role in the evolution of the language faculty (Kirby and Hurford, 1997; Jackendoff, 2002; Turkel, 2002). However, Chater et al. (2009) argue that the fact that languages change over time makes the situation of language evolution quite different from that of other learned traits. In their simulations, if the rate of language change is high enough, it is impossible for genetic evolution to keep up–language presents a moving target, and domain-specific constraints cannot evolve.

Chater et al.'s (2009) model is a critique of a particular view of the language faculty in which hard innate constraints are placed on the form languages can take. Because of this they do not model a scenario in which the strength of bias is allowed to evolve freely (although they do show that their model gives similar results whether genes encode hard constraints, or very strong biases). However, there is growing support for a more nuanced view of language acquisition in which learners have biases that come in a range of strengths (e.g., Morgan et al., 1989; Wilson, 2006; Hudson Kam and Newport, 2009; Smith and Wonnacott, 2010; Culbertson and Smolensky, 2012; Culbertson et al., 2013; Chater et al., 2015). If the genes underpinning the language faculty were able to specify everything from a very weak bias all the way to a hard constraint, then perhaps this would allow evolution to take a gradual path from an unbiased learner to a strongly-constraining, domain-specific language faculty. To find out if this is the case, we need a model that shows how bias strength affects the nature of the languages that emerge in a population.

**Figure 1:**
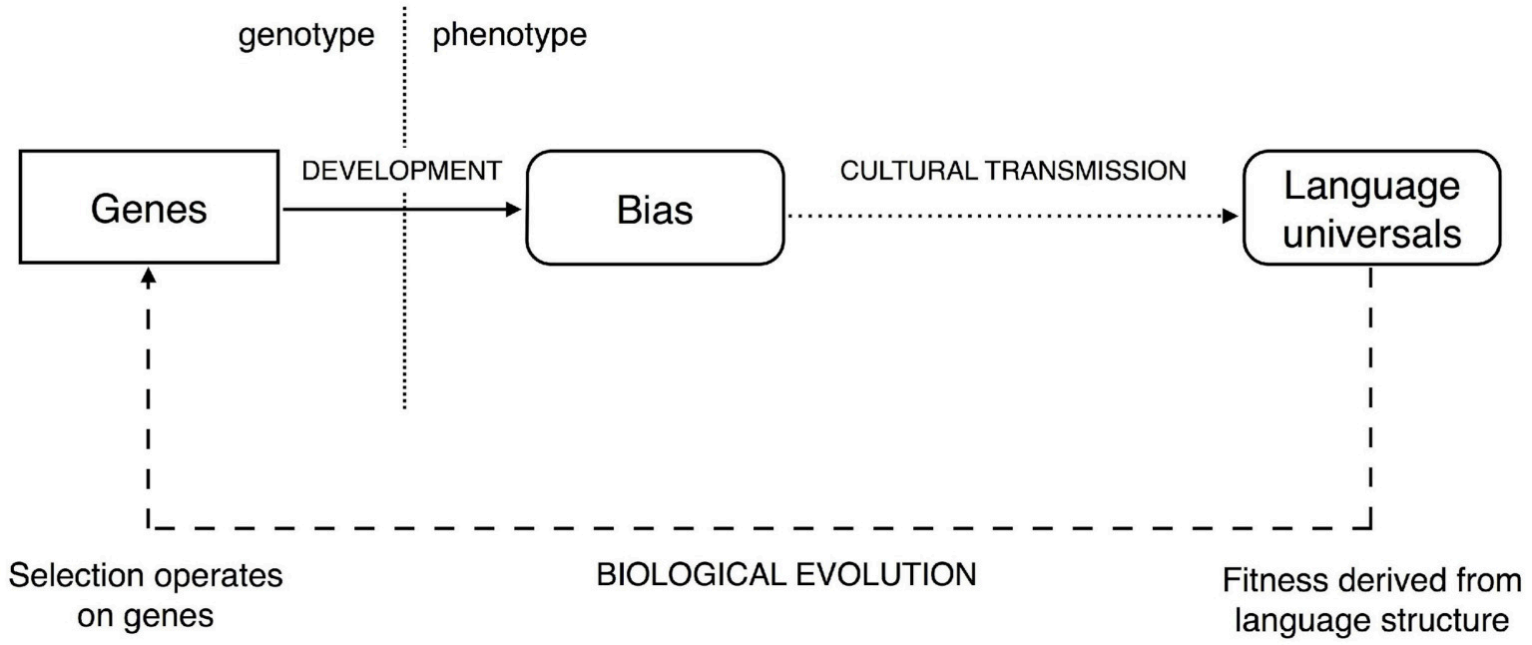
**The link between genes and the universal properties of language is mediated by development and cultural transmission**. The extent to which these two processes have non-trivial dynamics is an important consideration when proposing evolutionary accounts of language. Fitness does not depend directly on the genes underpinning the language faculty, but rather the linguistic phenotype (i.e., languages). This opens up the possibility for development and cultural transmission to shield genetic variation from the view of natural selection (Figure adapted from Kirby et al., 2007). © 2007 by The National Academy of Sciences of the USA.

The iterated learning model (Kirby et al., 2007) starts from the observation that the way languages evolve culturally is driven by the way in which languages are learned^2^. This model of cultural evolution suggests that the languages spoken by a population will not necessarily directly reflect the learning biases of that population (Figure 1). In particular, in many cases, cultural evolution will tend to amplify weak learning biases. This has important implications for how constraints on the language faculty actually come to be reflected in properties of language. For example, the observation that some property of language is universally, or near universally, present in language is not sufficient for us to infer that there is a corresponding strong constraint in our language faculty. Indeed, if Kirby et al. (2007) are correct, then the strength of any constraint in the language faculty may be *unrelated* to the strength of reflection of that constraint cross-linguistically. Weak learning biases may be sufficient to give rise to exceptionless, or near exceptionless, universals.

Smith and Kirby (2008) examine the implications of iterated learning for the biological evolution of the language faculty. Their simulation explicitly models three processes involved in the origins of linguistic structure: individual learning of languages from data; cultural evolution of languages in a population through iterated learning; and biological evolution of learning biases themselves. They show that neither hard constraints nor strong biases emerge from the evolutionary process even when agents are being selected for their ability to communicate using a shared language. This is a consequence of the amplifying effect of cultural evolution; the fitness of an organism is not derived directly from that organism's genes, but rather from the organism's phenotype. In the case of language evolution this is the actual language an individual has learned. If weak learning biases are amplified by cultural evolution, then the difference between a weak bias and a hard constraint is neutralized: both can lead to strong effects on the distribution of languages. What this means is that iterated learning effectively masks the genes underpinning the language faculty from the view of natural selection. They are free to drift; strongly-constraining domain-specific constraints on language learning are likely to be lost due to mutation, or not arise in the first place (see also, Thompson, 2015 for a detailed analysis of the evolutionary dynamics in this case).

Taken together these modeling results show that domain-specific hard constraints on language learning are unlikely to evolve, because languages change too fast (Chater et al., 2009) and because cultural evolution amplifies the effect of weak biases (Kirby et al., 2007). However, the results of this latter model suggest a further conclusion: weak biases for language learning are *more* evolvable by virtue of cultural evolution's amplifying effect. Any tiny change from neutrality in learning can lead to big changes in the language that the population uses. Just as culture masks the strength of bias from the view of natural selection, it unmasks non-neutrality. We argue that linguists should not shy away from formulating domain-specific aspects of the language faculty in terms of weak, defeasible biases. This is the type of language faculty that is most likely to evolve.

Although we propose that strong domain-specific biases on language should be avoided on evolutionary grounds, this does not mean that strong domain-*general* biases are impossible. These may be the result of very general architectural or computational considerations that govern the way cognition operates, for example (falling under the third of Chomsky's, 2005 three factors in language design). Equally, the way we learn language might be shaped by relatively strong domain-general biases that arise as a result of evolution for something other than language, for which the amplifying effect of culture does not apply. Biases such as these may nevertheless interact with language and linguistic representations in domain-specific ways. In the next section we will examine a learning bias that is arguably the most domain-general of all—simplicity—and show how its application in a range of different aspects of language leads to domain-specific outcomes.

## Simplicity

Simplicity has been proposed as a unifying principle of cognitive science (Chater and Vitányi, 2003). The tradition of arguing for a general simplicity bias has a long history in the context of scientific reasoning dating back to William of Occam in the 14th century who stated that we should prefer the simplest explanation for some phenomenon all other things being equal. In other words, when choosing among hypotheses that explain data equally well, the simpler one should be chosen.

**Figure 2:**
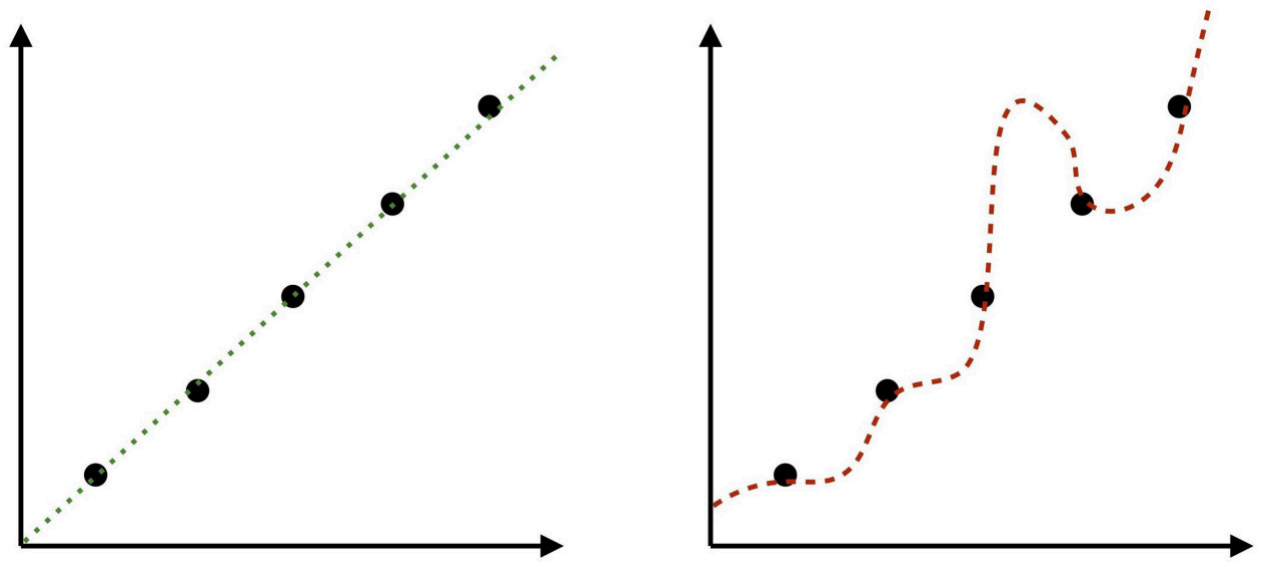
**There are an infinite set of possible functions interpolating from seen points to unseen points in these graphs**. Our intuition is that the linear function on the left represents a more reasonable hypothesis than the one on the right, despite the fact that both fit the data perfectly well. In other words, we have prior expectations about what functions are more likely than others. In this case, the prior includes a preference for linearity (cf. Kalish et al., 2007).

This principle can be extended straightforwardly from scientific reasoning to cognitive systems. When faced with an induction problem we must have some way of dealing with the fact that there are many candidate hypotheses that are consistent with the observed data (typically an infinite number). So, for example, in a function learning task how do we interpolate from seen to unseen points when there are an infinite number of possible functions that could relate the two (Figure 2). Or, to give a more trivial example, why is it that we assume that the sun will continue to rise every day when there are an infinite range of hypotheses available to us which predict it won't.

Here again the simplicity bias provides an answer by giving us a way to distinguish between otherwise equally explanatory hypotheses. While a full treatment of why simplicity rather than some other bias is the correct way to solve this problem is beyond the scope of this article (accessible introductions are given in Mitchell, 1997; Chater et al., 2015), we can give an intuitive flavor in terms of Bayesian inference. According to Bayes rule, induction involves combining the probability distribution over hypotheses defined by the data with a *prior* probability distribution over these hypotheses. More formally, the best hypothesis, *h*, for some data, *D*, will maximize *P*(*D*|*h*)*P*(*h*).

$$\begin{array}{l} h_{best}=argmax_{h\in H}P\left(h|D\right)=argmax_{h\in H}P\left(D|h\right)P\left(h\right) \end{array}$$

What can this tell us about simplicity? We can express this equivalently by taking logs of these probabilities. The best hypothesis is the one that *minimizes* the sum of negative log probabilities of the data given that hypothesis, −*log*_2_*P*(*D*|*h*), and the prior probability of the hypothesis itself, −*log*_2_*P*(*h*).

$$\begin{array}{l} h_{best}=argmin_{h\in H}-log_{2}P\left(h|D\right)\\ =argmin_{h\in H}-log_{2}P\left(D|h\right)-log_{2}P\left(h\right) \end{array}$$

Information theory (Shannon and Weaver, 1949) tells us that this last quantity, −*log*_2_*P*(*h*), is the description length of *h* in bits (assuming an optimal encoding scheme for our space of hypotheses). So, all other things being equal, learners will choose hypotheses that can be described more concisely—hypotheses that are simpler.

Importantly, an information theoretic view of the equation above also suggests learners will prefer representations that provide (to a greater or lesser extent) some compression of the data they have seen. What does this mean for the nature of language? It suggests that languages will be more prevalent to the extent that they are compressible. In general, a language will be compressible if there are patterns within the set of sentences of that language that can be captured by a grammatical description. More precisely, a compressible set of sentences is one whose minimum description length is short. The description length is simply the sum of the length of the grammar (−*log*_2_*P*(*h*) in the equation above) and the length of the data when described using that grammar (given by the −*log*_2_*P*(*D*|*h*) term).

This argument has allowed us to relate our intuitive understanding of simplicity—as a reasonable heuristic in choosing between explanations—to a rational model of statistical inference in a relatively straightforward way. Of course, there are a lot of practical questions that this leaves unanswered. How, for example, can we tell in a given domain what counts as a simpler hypothesis? Unfortunately, there is no computable general measure of complexity (Li and Vitányi, 1997), nevertheless we propose that notions of relative simplicity should guide our search for domain-general biases underpinning phenomena of interest in language.

So, we argue that—whatever other biases learners have when they face some learning problem—they are also likely to be applying an overarching simplicity bias (Chomsky, 1957; Clark, 2001; Brighton, 2002; Kemp and Regier, 2012; Chater et al., 2015).

It is important to note that when we talk about simplicity in the context of language, it is in terms of the overall compressibility of that language, e.g., how much redundancy and systematicity does it exhibit that can be captured simply in a grammatical description, and how much irreducible unpredictability remains in the data. We might also be interested in ways in which languages differ in the length of their utterances, but this is a largely orthogonal issue. Indeed, it is possible for a language with shorter strings to have a longer grammar—consider cases of irregular morphology in which regularization might simplify a paradigm at the cost of removal of short irregulars.

The generality of the bias for simplicity suggests there will be many linguistic phenomena affected by it. Below, we discuss cases which have been documented both in linguistic typological *and* experimental studies, with an emphasis on morphology and syntax (for discussion of experimental findings related to phonological simplicity, see Moreton and Pater, 2012a,b). We will begin with a basic design feature of language—compositionality—that can be characterized by the interaction of simplicity with a competing pressures for expressivity. We then move on to three additional examples of increasingly narrow phenomena: regularization of unconditioned variation, consistent head ordering or word order harmony, and isomorphic mapping from semantic structure to linear order. Each example illustrates a slightly different way in which this domain-general bias interacts with features that are particular to the linguistic domain.

### Compositionality

For our first example we will consider a basic property of language, often called a “design feature” (Hockett, 1960): the compositional nature of the mapping between meanings and forms. Language is arguably unique among naturally occurring communication systems in consisting of utterances whose meaning is a function of the meaning of its sub-parts and the way they are put together. For example, the meaning of the word “stars” is derived from the meaning of the root *star* combined with the meaning of the plural morpheme *-s*. Similarly, the meaning of a larger unit like “visible stars” is a function of the meanings of the individual parts of the phrase. Switching the order to “stars visible” changes the meaning of the unit in a predictable way^3^.

This ubiquitous feature of language makes it arguably unique among naturally occurring communication systems, the vast majority—perhaps all—of which are *holistic* rather than compositional (Smith and Kirby, 2012). The striking divergence from holism that we see in language (above the level of the word) is therefore of great interest to those studying the evolution of language. The fact that human communication is also highly unusual in consisting of learned rather than innate mappings between meanings and signals suggests that relating the origins of compositionality to learning biases is a good place to start in the search for an explanation.

**Figure 3:**
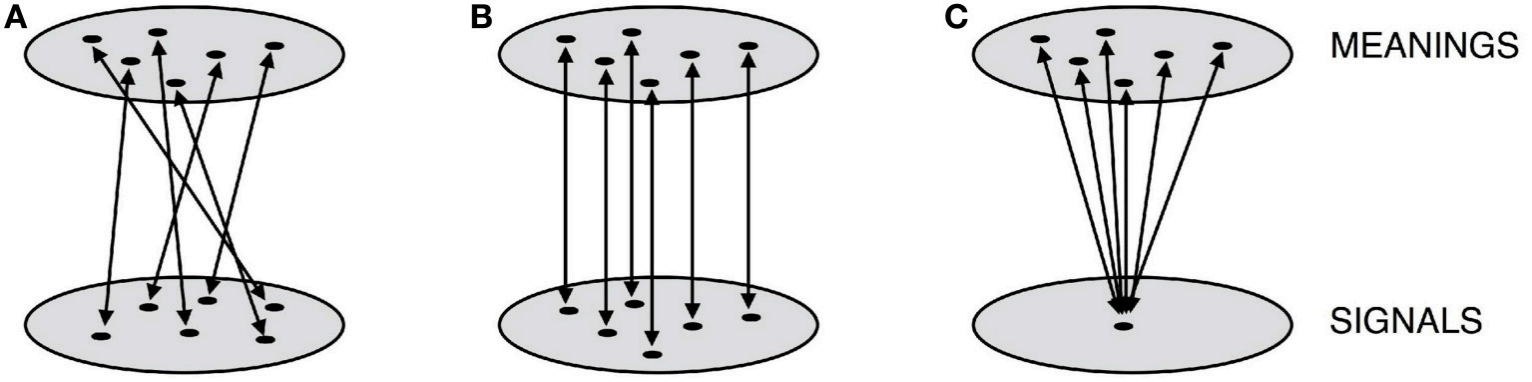
**A simplified geometric sketch of possible mappings between two domains, for example meanings and signals**. These mappings can be unstructured, random and incompressible **(A)**, or highly structured and compressible **(B)**. An individual attempting to learn the latter could use similarity structure in one domain to predict what the appropriate generalization should be for unseen points. A further possibility is a degenerate mapping, which is the simplest and most compressible of all **(C)**.

**Figure 4:**
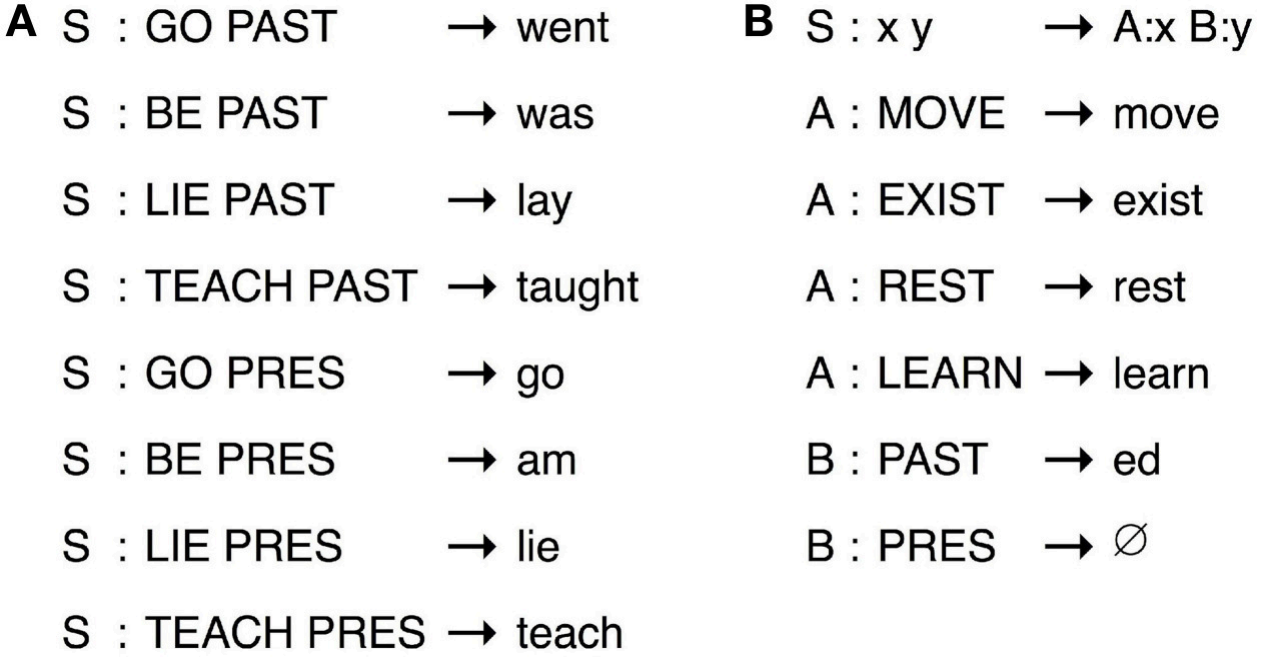
**Two simple transducers that map between a subset of the English verbs and their meanings, where “S” is the start symbol for the transducers and meanings are given in caps after a colon in each rule**. Transducers can be *holistic*, essentially a dictionary of meaning-signal pairs **(A)**; or *compositional*, in which the meaning of a signal is composed of the meaning of parts of that signal **(B)**.

A language that maps meanings onto signals randomly (see Figure 3A) will be less compressible—and hence, less *simple* in our terms—than one which maps them onto signals in a predictable way (see Figure 3B). Where both signals and meanings have internal, recombinable structure, then this predictability will be realized as compositional mappings. To see why this is, consider representing language as a *transducer* relating meanings and signals. The transducer in Figure 4A gives the most concise representation of an example holistic language, whereas the transducer in Figure 4B gives the most concise representation of an equivalent compositional language in which subparts of the signals map onto subparts of the meanings. What should be immediately apparent is that compositional languages are more compressible.

Brighton (2002) uses this contrast to model the cultural evolution of compositionality in an iterated learning framework (Kirby et al., 2007). Individual agents in their simulation learn transducers to map between a structured set of meanings and signals made up of sequences of elements. Crucially, the learners have a prior bias in favor of simpler transducers. In fact, the prior probability of a particular transducer is inversely related to its coding length in bits in precisely the way outlined in our discussion of simplicity above. Each agent learns their language by observing meaning-signal pairs produced by the previous agent in the simulation, and then goes on to produce meaning-signal pairs for transmission to the next generation. As the language in these simulations is repeatedly learned and reproduced, the bias of the agents in favor of simplicity shapes the evolutionary dynamic. Despite the fact that these models involve no biological evolution, the grammars adapt gradually over cultural generations from ones that are random and holistic to ones that are compositional^4^.

This result makes intuitive sense if you think about the process of transmission from the point of view of the emerging rules and regularities in the mapping between meanings and signals. A highly specific feature of the evolving language (e.g., a particular idiosyncratic label for a single meaning, like *went* as the past tense of GO) will be harder to learn than a generalization over a large number of meanings (e.g., a morpheme, like –*ed*, that shows up in the signals associated with a wide range of meanings). Particularly if learners only see a subset of all possible meanings, this inevitably leads to a preferential transmission of broader and broader generalizations that apply across large parts of the language. Hurford (2000) puts it pithily, stating “social transmission favors linguistic generalization.”

The simplicity bias thus appears to predict one of the fundamental design features of human language. However, things are not quite so straightforward. Consider a language in which every meaning is expressed by the same signal (Figure 3C). This *degenerate* language will be even more compressible than the compositional one, suggesting that a domain-general bias for simplicity is not sufficient to explain the origins of compositional structure. Cornish (2011) argues that in fact all simulations of iterated learning purporting to demonstrate the emergence of compositionality have in some way implemented a constraint that rules out degeneracy. It is simply impossible for the learners in these simulation models to acquire a language that maps many meanings to one signal. Similarly, in the first laboratory analog of these iterated learning simulations, Kirby et al. (2008) report that degenerate languages rapidly evolve over a few generations of human learners.

Kirby et al. (2015) argue that a countervailing pressure for expressivity is required to avoid the collapse of languages in iterated learning experiments to this degenerate end point. The obvious pressure arises not from learning, but from use. If pairs of participants learn an artificial language and then go on to use it in a dyadic interaction task, then there are two pressures on the language in the experiment: a pressure to be compressible arising from participants' domain-general simplicity bias in learning, and a pressure to be expressive arising from participants' use of the language to solve a communicative task. Kirby et al. (2015) show that compositionality only arises when both of these two pressures are in play. In this case then, a domain-general bias is only explanatorily adequate once we take into account features of its domain of application. In other words, the case of compositionality illustrates that the simplicity bias is domain-specific in the sense that we cannot understand how it shapes language without also appealing to the special function of language as a system of communication.

### Regularization

There is converging evidence from multiple strands of research including pidgin/creole studies, sociolinguistics, language acquisition, and computational cognitive science suggesting that language tends to minimize unpredictable or unconditioned variation. Variation can be introduced by non-native speaker errors, contact with speakers of other languages, or in the case of newly emerging languages, variation may reflect a lack of conventionalized grammar. In the latter case, there is evidence that new generations of learners regularize and conventionalize these noisy systems (e.g., Sankoff, 1979; Mühlhäusler, 1986; Meyerhoff, 2000; Senghas and Coppola, 2001). Natural language and laboratory language learning research has further shown that both children and adults learn and reproduce conditioned variation relatively well compared to unpredictable variation (e.g., Singleton and Newport, 2004; Hudson Kam and Newport, 2005, 2009; Smith et al., 2007; Smith and Wonnacott, 2010; Culbertson et al., 2012). For example, Singleton and Newport (2004) report the case of a child acquiring American Sign Language (ASL) from late-learner parents. While the parents' realization of several grammatical features of ASL was variable, the child did not reproduce this variation. Rather, he regularized his parents' variable productions, resulting in a much more consistent system (though in some aspects it differed from ASL). Following up on this finding using an experimental paradigm, Hudson Kam and Newport (2009) report that, when trained on a grammar with unpredictable use of determiners, child learners (and to a lesser extent adults) regularize those determiners, using them according to a consistent rule.

Computational modeling has formalized this in terms of learners' a priori expectations, namely that observed data come from a deterministic generative process (Reali and Griffiths, 2009; Culbertson and Smolensky, 2012; Culbertson et al., 2013). This has a natural interpretation in terms of simplicity, since the description of a language that only allows one option in a particular context will be shorter than one that allows multiple variants^5^. More generally, as we've seen already, there's a straightforward relationship between the entropy of the distribution of variants and the coding length of that distribution. More predictable processes can be captured by shorter overall descriptions: they are compressible (Ferdinand, 2015). However, the expectation that the world will be deterministic is to some extent dependent on the domain in question. Most obviously, prior experience in a given domain can override this expectation—e.g., we expect that a coin being tossed will be fair and therefore outcomes will be random (Reali and Griffiths, 2009). In a carefully controlled study comparing learning of unpredictable variation in a linguistic vs. non-linguistic domain, Ferdinand (2015) found that regularization occurs in both domains. However, across a number of conditions manipulating system complexity, the bias is stronger for linguistic stimuli. Regularization thus illustrates a case in which the *strength* of a bias is domain-specific, perhaps dependent on previous experience and functional pressures relevant to that domain.

While most recent work on regularization focuses on unconditioned or random variation, there is some evidence that even conditioned variation is avoided in language. For example, English is losing its system of irregular (variable) past tense marking in favor of a single rule (add *-ed*) despite this variation being lexically conditioned (Hooper, 1976). Similarly, while some languages allow widespread lexically or semantically conditioned variation in adjective placement, most languages tend to order them more or less consistently before or after (Dryer, 2013). This can be related straightforwardly to simplicity; a grammar with a single (high-level) rule or constraint applying to all words of a given type is more compressible than one in which different such words must obey different rules. For example, a grammar with a single rule stating that adjectives must always precede nouns is simpler than one which has to specify that certain adjectives precede and others follow.

### Harmony

Interestingly, this reflex of simplicity applies not only to word order within a word class, but also across classes of words. Some of the best known typological universals describe correlations among words orders across different phrase types. For example, Greenberg (1963) lists a set of universals, collated from a sample of 30 languages, including the following:

*Universal 2*: In languages with prepositions, the genitive almost always follows the governing noun, while in languages with postpositions it almost always precedes.
*Universal 18:* When the descriptive adjective precedes the noun, the demonstrative and the numeral, with overwhelmingly more than chance frequency, do likewise.

These universals are part of the evidence for word order harmony—the tendency for a certain class of words to appear in a consistent position, either first or last, across different phrase types in a given language (Greenberg, 1963; Chomsky, 1981; Hawkins, 1983; Travis, 1984; Dryer, 1992; Baker, 2001; for experimental evidence see Culbertson et al., 2012; Culbertson and Newport, 2015). At its root, this is just an extension of the same very general statement of within-category order consistency. However, absent a notion of what ties certain categories of words together, the connection between harmony and simplicity remains opaque. For example, the two universals quoted above make reference to a single category—noun—and how it is ordered relative to a number of other categories. Based on syntactic class alone, simplicity predicts that nouns should be ordered consistently relative to all these other categories. This is, of course, the wrong prediction; Universal 2 actually says that the order of nouns relative to adpositions is the *opposite* of the order of nouns relative to genitives. While adpositions and genitives thus tend to appear on different sides of the noun, it turns out that adjectives, demonstratives, and numerals often pattern with genitives (note that English is a counterexample). These tendencies are exemplified in (3).

3) a. Preposition N {Adj, Num, Dem, Gen}
     b. {Adj, Num, Dem, Gen} N Postposition

To make sense of this, we need a notion that connects adpositions as they relate to nouns, with nouns as they relate to the other categories. The most popular such notion provided by linguistic theory is the head-dependent relation. In this example, the noun is a head with respect to nominal modifiers—including genitive phrases, adjectives, numerals, and demonstratives. By contrast, the noun is a dependent in an adpositional construction. When stated in this way, harmony falls out: in the world's languages, there is a tendency for heads to consistently precede *or* follow their dependents. The former type is often called head-initial, the latter head-final. Coming back to simplicity then, a language which has a single high-level rule stating that heads either precede or follow their dependents is simpler than one which has specific ordering rules for heads in distinct phrase types. Simplicity therefore predicts that the more specific rules a grammar has, the less likely it should be.

Importantly, a clear understanding of whether this prediction is borne out depends on the precise definition of the relevant relation between word categories. This turns out to be controversial. For example, particular theories differ in what is deemed to be a head, and whether “head” is in fact the relevant notion at all (Hawkins, 1983; Zwicky, 1985; Hudson, 1987; Dryer, 1992; Corbett et al., 1993). Dryer (1992) provides typological evidence that head order does not correlate across all phrase types. For example, he reports that the order of verb (head) and object (dependent) correlates with the order of preposition (head) and noun (dependent) within a language, but not with noun (head) and adjective (dependent) order. This is unexpected if the simplicity bias is indeed based on head-dependent order. He therefore argues that a different notion, related to the average length or complexity of particular phrase types, must be used in order to see that languages do indeed prefer higher-level rules governing order across multiple phrase types. Regardless of whether Dryer's precise formulation is correct, what this suggests is that merely stating that simplicity is a factor in determining word order does not allow us to determine which grammars are in fact the simplest. In order to do this, we need a theory of linguistic representations which tells us which should be treated as parallel and in what contexts.

From the perspective of the learner, there is also a clear sense in which the simplicity bias as it relates to word order harmony depends on linguistic representations. Given three words, in the absence of any knowledge about the relations between and among them, there is no way simplicity can be used by a learner to make inferences about likely orderings. These representations must be present (e.g., learned) before a simplicity bias can be active. How and when they develop—i.e., when particular syntactic categories are differentiated, when abstract higher-level categories like head develop, etc.—will dictate how simplicity impacts learners' inferences.

### Isomorphic mapping

The relation between word order and semantic interpretation in a number of domains also appears to be affected by a simplicity bias. For example, Greenberg's (1963) Universal 18 describes how nominal modifiers are ordered relative to the noun. Universal 20 builds on this, describing how those modifiers tend to be ordered relative to one another.

*Universal 20* (as restated by Cinque, 2005)*:*
In pre-nominal position the order of demonstrative, numeral, and adjective (or any subset thereof) is *Dem-Num-Adj*.
In post-nominal position the order is either *Dem-Num-Adj* or *Adj-Num-Dem*.

Interestingly, while both post-nominal orders are indeed possible, addition typological work since Greenberg (1963) indicates that the second order is *much* more common. In fact, Dem-Num-Adj-N, and N-Adj-Num-Dem are the two most common orders found in the world's languages by far. Part of this is likely due to the harmony bias described above; assuming nominal modifiers are covered by the relevant notion of dependent, these two orders are harmonic, while alternative possibilities are not (e.g., Dem-Num-N-Adj). However, harmony does not explain why N-Adj-Num-Dem would be more common than N-Dem-Num-Adj. An explanation of this difference depends on how syntax–specifically, linearization—interacts with underlying semantic structure.

**Figure 5:**
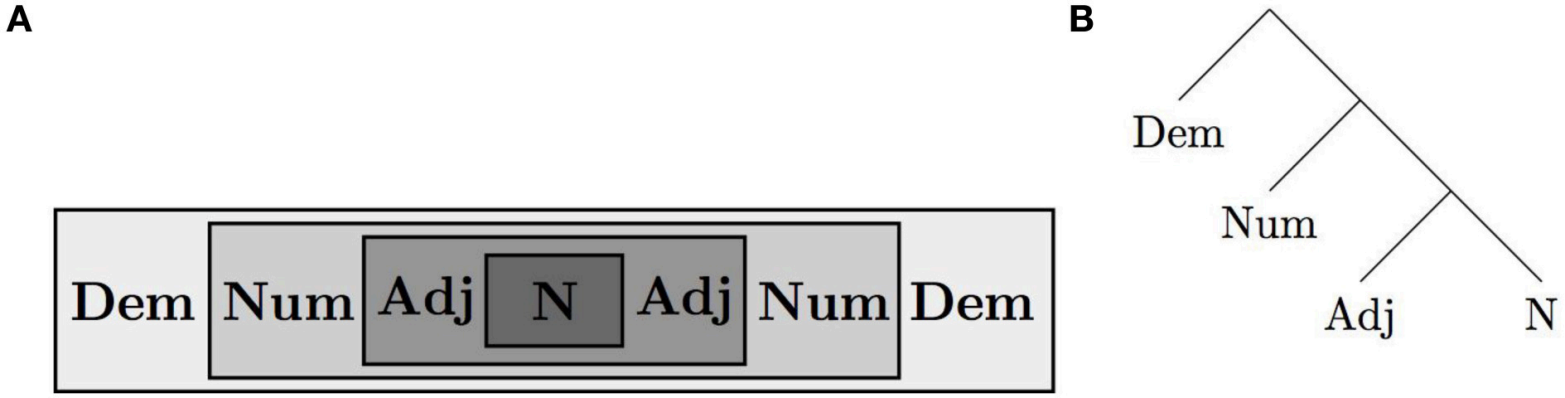
**Nested representation (A) and hierarchical representation (B) of semantic relations between modifiers and the noun**. The most typologically common orders can be read off directly.

Several theoretical lines of research converge on a universal semantic representation of these modifiers and their relation to the noun. On one view, this representation reflects iconicity of relations (Rijkhoff, 2004). For example, adjectives modify inherent properties of nouns, numerals count those larger units, and demonstratives connect those countable units to the surrounding discourse. This describes a nesting representation as in Figure 5A. Research in formal linguistics further suggests a hierarchical relation between these elements in terms of semantic combination, illustrated in Figure 5B. Crucially, these abstract relations are preserved in linear orders that have the adjective closest to the noun and the demonstrative most peripheral—orders that can be read directly off Figure 5A. Notice that N-Adj-Num-Dem is one such order, while N-Dem-Num-Adj is not (the modifiers must be swapped around to get this order). Recent laboratory studies suggest a corresponding cognitive bias, in favor of isomorphic mappings between nominal semantics and linear order (Culbertson and Adger, 2014). Typological frequency differences in this domain can be therefore be much better explained once we take into account the underlying semantic structure and an isomorphism bias.

**Figure 6:**
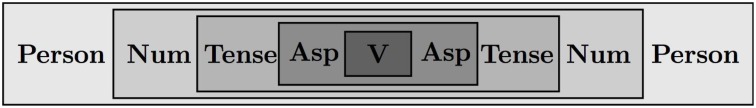
**Schematic representation of semantic composition in verbal domain**.

This is not the only case of isomorphic mappings from semantics to linear order, indeed perhaps the most well-known case is the mirror principle in the domain of verbal inflection (Baker, 1985; Bybee, 1985; Rice, 2000). Languages tend to order inflectional morphemes like tense and aspect in a way that reflects semantic composition, as shown in Figure 6,^6^.

Biases in favor of isomorphism between semantics and linear order can again be reduced to a general simplicity bias. In very general terms, more transparent or predictable relations between order and meaning are simpler than ones with extra arbitrary stipulations. Brighton and Kirby (2006) show that isomorphic^7^ mappings between signals and meanings arise naturally from iterated learning under general simplicity considerations. Put in more precise terms, to derive surface order from semantics, each branch of the hierarchical structure (or each rectangle in the nested schematic) in the figure above represents a choice point for linearization. For isomorphic orders, that is all that is required: N-Adj-Num-Dem means choosing (1) Adj after N, (2) Num after [N-Adj], and (3) Dem after [N-Adj-Num]. Similarly, a non-harmonic but isomorphic order like Dem-Num-N-Adj is (1) Adj after N, (2) Num before [N-Adj], and (3) Dem before [Num-N-Adj]. By contrast, non-isomorphic orders require additional choice points or rules. N-Dem-Num-Adj, for example, cannot be derived from the semantic hierarchy alone—the simplest route is Dem-Num-Adj-N (three choice points) plus one addition rule placing N first. The isomorphism bias again illustrates that the notion of simplicity, however general, must be formulated with reference to specific hypotheses about the domain in question—here, about conceptual iconicity or formal compositional semantics.

## Conclusion

There is little doubt that the language faculty includes capacities and constraints that are domain-general or co-opted from other cognitive systems. Whether it also includes domain-specific features is both less clear, and more likely to split along philosophical lines; traditionally, generative linguistics has argued for a Universal Grammar containing (among other things) linguistically contentful principles that place hard constraints on what is learnable. We have suggested, based on results obtained using computational models of language evolution, that domain-specific hard constraints are much less likely to have evolved than weak biases. This is essentially because the cultural evolution of language exerts cognition-external pressures that mean linguistic phenotypes no longer directly reflect the underlying genotype. The strength of any particular bias is underdetermined by the cross-linguistic distribution of language types. At the same time, these cognition-external pressures allow weak genetically-encoded biases to have potentially large typological effects. While this does not categorically rule out the existence of very strong (or inviolable) biases that have evolved specifically for language, it clearly suggests we should not treat them as the default hypothesis. The idea that weak biases for language-specific structures or patterns are more likely is in line with recent trends in linguistics. Researchers in phonology and syntax have begun using formal models which encode probabilistic biases in order to better capture empirical data from typology and learning (e.g., Hayes and Wilson, 2008; Pater, 2009; Culbertson et al., 2013; White, 2014).

Regardless of whether the language faculty contains domain-specific capacities, the representations which make up our linguistic knowledge, and the function of language as a system of communication means that domain-*general* capacities will interact with language in unique ways. This is most convincingly illustrated by looking at an uncontroversially general bias: the bias in favor of representational simplicity. The examples we have discussed here show that a simplicity bias is reflected in a range of language universals that cut across very different aspects of the linguistic system: compositionality, regularity, harmony, and isomorphism. In each case, the simplicity bias interacts with linguistic representations to give rise to domain-specific effects. In the case of compositionality, simplicity interacts with the major unique function of language as a communication system that must be expressive. It is only via the interaction of these two pressures that compositional systems will emerge. The regularization bias, which describes the established finding that language learners tend to reduce random or unconditioned variation, shows domain-specific effects in terms of its strength. Word order harmony, the tendency for languages to order heads consistently before or after dependents, depends crucially on a language- and even theory-specific notion of the relevant categories. Finally, the notion of isomorphism between semantic or conceptual structure and surface word order crucially requires an articulated hypothesis about the specific semantic relations among dependent elements.

In all these cases, distinct hypotheses about linguistic categories, their representations, and how they relate to one another will make distinct predictions about how simplicity is cashed out. This means that an understanding of language, how it is learned, and how it evolved will necessarily require input from linguists formulating theories of the architecture and representations of language. The fact the many aspects of the capacity for language also come from broader cognition means linguists in turn must take into account findings from research on other cognitive domains, and indeed on related capacities in other species.

### Conflict of interest statement

The authors declare that the research was conducted in the absence of any commercial or financial relationships that could be construed as a potential conflict of interest.

## Footnotes

^1^Note this is true even if we then happen to use this aspect of our cognitive system for other, additional purposes. The fact that we use our language faculty for solving crosswords does not constitute an argument against domain-specificity of that faculty.

^2^Our emphasis in this article will be on learning, but there are other mechanisms that operate at the individual level but whose effect is felt at the population level. For example, the way in which hearers process input, and the way in which speakers produce output is likely to have a significant impact. See Kirby (1999) for an extended treatment of precisely how processing and learning interact with cultural transmission to give rise to language universals, and Futrell et al. (2015), Fedzechkina et al. (2012), and Jaeger and Tily (2011) for recent accounts of specific links between processing and language structure. However, the debate about domain generality/specificity plays out differently for processing than for learning, and as such will not be the focus of this review. In particular, here we discuss *simplicity* as a highly general learning bias that unifies a range of different domains both within and beyond language, and it is not clear that an equivalent notion of simplicity exists for processing.

^3^In this case, placing the adjective after the noun leads to the interpretation “the stars visible (tonight).” This is a systematic rule of English: post-nominal attributive adjectives are stage-level predicates, denoting temporary properties (Cinque, 1993).

^4^Brighton (2002) makes the simplicity bias of the learners in his model overt by counting the numbers of bits in the encoding of transducers that generate the data the learners see. However, this does not mean that we necessarily believe that this kind of representation of grammars is necessary for an implementational or algorithmic account of what language we are doing when they learn language. Rather, this is a computational level account in (Marr, 1982) terms. It is an empirical question whether the particular ranking of grammars in terms of simplicity that we can derive from this particular representation matches precisely the ranking that applies in the case of real language learners, but we are confident that the crucial distinction between degenerate < compositional < holistic is correct. This matches behavior of participants in the lab (Kirby et al., 2015) and broadly similar results are found in both connectionist and symbolic models of iterated learning (Kirby and Hurford, 2002; Brace et al., 2015).

^5^Note that this requires taking into account the simplicity of the generating grammar *and* the simplicity (compressibility) of the data. A grammar which allows free variation may be simpler than a grammar which generates conditioned variation, however the random data produced by the former grammar is not compressible.

^6^Interestingly, the acquisition of semantics literature provides a related observation. Musolino et al. (2000) show that when asked to interpret ambiguous sentences with quantificational elements, children strongly prefer the interpretation that corresponds to the surface syntactic position of those elements. For example, the sentence “Every horse didn't jump over the fence,” could involve *every* taking scope over *not* (meaning no horses jumped over the fence), or *not* scoping over *every* (meaning not every horse jumped over the fence). The first interpretation is isomorphic to the linear order, and this is the interpretation preferred by young children (see also Musolino and Lidz, 2003).

^7^These authors use the term “topographic” rather than “isomorphic” because of similarity to the neuroanatomical organizing principle of topographic maps. For our purposes the terms are interchangeable, since both give rise to the property that neighboring representations in one domain map to neighboring representations in the other.
